# Untargeted Lipidomic Biomarkers for Liver Cancer Diagnosis: A Tree-Based Machine Learning Model Enhanced by Explainable Artificial Intelligence

**DOI:** 10.3390/medicina61030405

**Published:** 2025-02-26

**Authors:** Cemil Colak, Fatma Hilal Yagin, Abdulmohsen Algarni, Ali Algarni, Fahaid Al-Hashem, Luca Paolo Ardigò

**Affiliations:** 1 Department of Biostatistics, and Medical Informatics, Faculty of Medicine, Inonu University, 44280 Malatya, Turkey; cemil.colak@inonu.edu.tr; 2 Department of Computer Science, King Khalid University, Abha 61421, Saudi Arabia; a.algarni@kku.edu.sa; 3 Department of Informatics and Computer Systems, College of Computer Science, King Khalid University, Abha 61421, Saudi Arabia; akafeer@kku.edu.sa; 4 Department of Physiology, College of Medicine, King Khalid University, Abha 61421, Saudi Arabia; fahaid999@yahoo.com; 5 Department of Teacher Education, NLA University College, Linstows Gate 3, 0166 Oslo, Norway

**Keywords:** liver cancer, lipidomics, biomarkers, machine learning, SHAP, precision medicine

## Abstract

*Background and Objectives*: Liver cancer ranks among the leading causes of cancer-related mortality, necessitating the development of novel diagnostic methods. Deregulated lipid metabolism, a hallmark of hepatocarcinogenesis, offers compelling prospects for biomarker identification. This study aims to employ explainable artificial intelligence (XAI) to identify lipidomic biomarkers for liver cancer and to develop a robust predictive model for early diagnosis. *Materials and Methods*: This study included 219 patients diagnosed with liver cancer and 219 healthy controls. Serum samples underwent untargeted lipidomic analysis with LC-QTOF-MS. Lipidomic data underwent univariate and multivariate analyses, including fold change (FC), *t*-tests, PLS-DA, and Elastic Network feature selection, to identify significant biomarker candidate lipids. Machine learning models (AdaBoost, Random Forest, Gradient Boosting) were developed and evaluated utilizing these biomarkers to differentiate liver cancer. The AUC metric was employed to identify the optimal predictive model, whereas SHAP was utilized to achieve interpretability of the model’s predictive decisions. *Results*: Notable alterations in lipid profiles were observed: decreased sphingomyelins (SM d39:2, SM d41:2) and increased fatty acids (FA 14:1, FA 22:2) and phosphatidylcholines (PC 34:1, PC 32:1). AdaBoost exhibited a superior classification performance, achieving an AUC of 0.875. SHAP identified PC 40:4 as the most efficacious lipid for model predictions. The SM d41:2 and SM d36:3 lipids were specifically associated with an increased risk of low-onset cancer and elevated levels of the PC 40:4 lipid. *Conclusions*: This study demonstrates that untargeted lipidomics, in conjunction with explainable artificial intelligence (XAI) and machine learning, may effectively identify biomarkers for the early detection of liver cancer. The results suggest that alterations in lipid metabolism are crucial to the progression of liver cancer and provide valuable insights for incorporating lipidomics into precision oncology.

## 1. Introduction

Liver cancer represents a significant global health burden, ranking as the sixth most common cancer and the third leading cause of cancer-related mortality worldwide. Hepatocellular carcinoma, representing the most common form of liver cancer, is often diagnosed at an advanced stage when very few options exist for effective treatment. Consequently, the five-year survival rate remains below 20% [1]. Identifying new therapeutic targets and early diagnosis are, therefore, of the utmost importance to help improve the prognosis of this fatal disease. In addition, emerging evidence has identified one of the most important biological processes underlying the development and progression of liver cancer as lipid metabolism. Lipids have active roles beyond structural building blocks in cellular membranes. They modulate essential biological processes, such as energy storage, cell signaling, and apoptosis. Cancer recognizes the dysregulation of lipid metabolism in tumor cells, known as ’lipid reprogramming’, as a hallmark. The reprogramming forms a basis that ensures a supply promoting tumor growth characterized by cellular proliferation, evasion of apoptosis, and also facilitating a microenvironment prone to metastasis [1,2,3].

Despite significant advancements in lipidomics, we still need to determine the dynamic nature of lipids before the onset of liver cancer. These early changes in lipid metabolism could be highly valuable as biomarkers because they give us early information about the molecular process that controls the development of hepatocarcinogenesis. If properly mapped out, this could pave the way for timely detection and treatment, which would benefit patients at risk. Indeed, combining untargeted lipidomics with sophisticated technologies like high-resolution mass spectrometry has presented significant opportunities in these areas. Thus, one can obtain the complete profile of various lipid species present in biological samples, enabling the identification of patterns or changes that would otherwise remain undetected. The analysis of these complex datasets will allow the researcher to specify those key lipids that will denote the early development stage of liver cancer. Previous studies centered their research on targeted lipidomics with smaller lipid panels. The current study adds value to prior research by uniting untargeted lipidomic analysis with XAI-driven machine learning systems to develop new diagnostic biomarkers. Our research advances beyond traditional approaches, which mainly used statistical methods, including logistic regression and PLS-DA, by integrating tree-based modeling with SHAP explainability for better biomarker identification and transparent modeling. These findings advance the understanding not only of how disturbed lipid metabolism may contribute to liver cancer but also of providing a promising basis for the development of new tools for the early detection of the disease. As research continues, the hope is that these insights will lead to more effective strategies for prevention and personalized treatment, bringing us closer to reducing the burden of liver cancer [4,5,6,7].

Improved computational methods, primarily machine learning and XAI, have revolutionized the field of lipidomics. Technologies are powerful tools to unravel the complexities of high-dimensional data, which typically overwhelm traditional methods. AdaBoost, Random Forest, and SHAP are among the outstanding representative techniques. While these methods are among the best for identifying critical biomarkers, they also provide further insight into the reasoning behind their predictions. This interpretability, particularly in the clinical setting, significantly transforms the landscape, as the decisions it informs often have significant consequences. By way of example, health professionals might understand, in detail, why a model classified a specific biomarker as relevant or just exactly why that model made its predictions. This transparency enhances the veracity of predictive models and makes them more practical for real-world implementation in areas, such as treatment guidelines and personalized medicine strategies. By bridging the gap between raw computing power and human understanding, these approaches work together in a way that is both scientifically sound and easy to understand. This makes sure that the insights gained from lipidomics research are not only cutting-edge but also usable, giving the clinician the power to make decisions for the patient that are based on knowledge and confidence [8,9,10].

The aim of this study was to identify biomarker candidate lipids that play a role in the development of liver cancer and develop an interpretable prediction model integrated with XAI to evaluate the potential of these lipids as early diagnosis biomarkers.

In this study, we have used untargeted lipidomics in pre-diagnostic serum samples from 219 liver cancer cases and 219 controls. We combined advanced profiling of lipidomics with machine learning and XAI techniques with the following focus:

1. Determine the lipid species substantially correlated with the risk of liver cancer;
2. Evaluate the value of these lipids as biomarkers for early detection;
3. Examination of the molecular mechanisms behind lipid reprogramming in liver cancer.

These findings provide new insight into the metabolic underpinning of liver cancer and prove the utility of lipidomics in conjunction with advanced computational methods for biomarker discovery and its clinical translation. A multidisciplinary approach for bridging gaps between basic tumor biology and precision medicine is fulfilling and will offer enhanced diagnostic and therapeutic possibilities.

## 2. Materials and Methods

### 2.1. Study Cohort and Laboratory Procedures

This study utilized publicly available lipidomic data obtained from the MetabolomicsWorkBench database. Blood samples from a case control study involving 219 liver cancer cases and 219 controls were obtained and analyzed based on high resolution mass spectrometry-based untargeted lipidomic analysis. Prospective volunteers who had any malignancy (except nonmelanoma skin cancer), advanced cirrhosis, chronic alcohol consumption, or other diseases that might limit participation, were excluded.

Controls were matched 1:1 to cases based on age at randomization (±5 years), date of blood collection (±30 days), the number of freeze–thaw cycles, and the laboratory in which any previous aliquoting was performed. Blood samples were obtained from participants during the baseline visit following an overnight fast, often lasting at least 12 h. Blood samples were centrifuged, and serum aliquots were collected and stored at −70 °C. Lipidome analysis was conducted utilizing 50 μL of serum. Complex lipids were assessed semiquantitatively by an untargeted method utilizing liquid chromatography coupled with quadrupole time-of-flight mass spectrometry (LC-QTOF-MS). Retention times were standardized via internal standards. After removing poorly detected and duplicate signals, 462 unique, annotated lipid species were identified. Raw peak heights were normalized through systematic error reduction using random forest (SERRF) to eliminate undesirable systematic variance. The principal lipid classes discussed encompass acylcarnitines (ACs), ceramides (CERs), cholesterol esters (CEs), diacylglycerols (DGs), fatty acids (FAs), lysophosphatidylcholines (LPCs), lysophosphatidylethanolamines (LPEs), plasmalogen phosphatidylcholines (plasmaPCs), plasmalogen phosphatidylethanolamines (plasmaPEs), phosphatidylcholines (PCs), phosphatidylethanolamines (PEs), phosphatidylinositols (PIs), sphingomyelins (SMs), and triacylglycerols (TAGs) [11].

### 2.2. Data Analysis

The lipidomic data were examined using univariate and multivariate statistical/machine learning methods. In the statistical method, the data were standardized using the median and then applied to the Pareto scale for multivariate analysis. The *t*-test was used to examine significant changes in lipidomics levels, and the false discovery rates (FDRs) were evaluated using the Benjamini–Hochberg approach to reduce the occurrence of false positives. Fold changes (FCs) were computed to assess disparities between lipids derived from liver cancer patients and lipids obtained from healthy patients. Significance was determined based on FDR-adjusted *p*-values of ≤0.05 and FCs of ≥1.2 for upregulated lipids or ≤1.2 for downregulated lipids. In addition, we employed the Volcano plot to visually represent the lipids that exhibited consistent up- or downregulation in liver cancer patients compared to healthy controls. PLS-DA analysis and Elastic Net feature selection were utilized independently to discover the lipid signature contributing to group discrimination and to evaluate the prediction ability of prospective biomarkers in distinguishing liver cancer. Subsequent examination was conducted using lipids shared among all three approaches (FC, PLS-DA Variable Importance in Projection-VIP Score, Elastic Net feature selection [12,13,14].

In multivariate analyses, predictive models were created using Adaptive Boosting (AdaBoost), random forest (RF), and gradient boosting algorithms to differentiate between liver cancer and healthy controls. These methods have demonstrated their resilience in handling data with many dimensions and are extensively utilized for analyzing different types of ‘omics’ data [13,15,16,17]. During the modeling phase, the patients were divided into a training set and a test set using stratified random sampling, with a ratio of 4:1. Stratified random sampling was employed to maintain class distribution across subsets. The performance of each model was assessed and contrasted on the test set. To improve the robustness of the prediction model, we performed the persistence technique 100 times using a variety of random seeds to avoid biased results and minimize overfitting and determined the average performance over these 100 iterations. The models’ performance was evaluated by calculating the area under the curve (AUC) with a 95% confidence interval and accuracy, F1 score, sensitivity, and specificity. SHAP, an XAI technique, was then used to provide clinical explanations for the algorithm’s decisions and to visually represent the impact of biomarker candidate lipids. The procedures used are described in the subsections.

### 2.3. Data Preparation and Normalization

Raw lipidomics data underwent an extensive preparation procedure before analysis. The initial phase involved the normalization of the data utilizing median values. The Pareto scaling approach was employed for multivariate studies. The standardization techniques enabled the comparability of lipid data across various sizes.

### 2.4. Power Analysis and Univariate Statistical Analysis

The MetSizeR package in the R program was used for sample size estimation for metabolomics analysis. A target false detection rate of 5% and an expected significant spectral splitting rate of 20% were used in the power analysis, along with an estimate of a minimum sample size of 100 subjects. Normality was confirmed via the Shapiro–Wilk test. An independent sample *t*-test was utilized to assess significant alterations in lipid levels. FDR values were computed employing the Benjamini–Hochberg method to reduce false positive outcomes. FC values were computed to ascertain lipid disparities between liver cancer patients and healthy control groups. Statistical significance was established using the criterion of FDR-corrected *p*-value < 0.05 and FC values ≥ 1.2 for upregulated lipids and ≤1.2 for downregulated lipids. Furthermore, Volcano plot analysis was used to visually depict lipids that were consistently upregulated or downregulated in liver cancer patients relative to controls.

### 2.5. Selection of Features

PLS-DA analysis and the Elastic Net feature selection approach were utilized independently to identify molecular signatures that differentiate across groups and assess prospective biomarkers’ prediction capability. Subsequent analyses included lipids common to all three methodologies (FC, PLS-DA VIP Score, Elastic Net feature selection). PLS-DA analysis was utilized to examine lipidomic data’s intricate structure and uncover chemical signatures that differentiate across groups. This technique concurrently executes dimensionality reduction and classification on high-dimensional data. VIP ratings were computed to assess the importance of each lipid in differentiating the groups. Metabolites with VIP scores exceeding 1.0 were deemed major contributors to group prejudice. Elastic Net feature selection was a regularization technique that amalgamates the benefits of L1 (Lasso) and L2 (Ridge) regression methodologies. This approach mitigates multicollinearity while simultaneously executing feature selection. In Elastic Net regression, compounds exhibiting nonzero coefficients were identified as possible biomarkers [18,19,20,21].

### 2.6. Construction of Machine Learning Models

Predictive models, including AdaBoost, Random Forest, and gradient boosting algorithms, were created to differentiate between liver cancer patients and healthy controls. These methods were favored for their robustness in handling high-dimensional data and extensive application in analyzing diverse ‘omics’ data. Patients were allocated into training and test sets during the modeling phase using a stratified random selection procedure at a 4:1 ratio. The AdaBoost technique relies on sequential training and the aggregation of weak classifiers, typically decision trees. In each iteration, model performance improved by assigning greater weight to the instances misclassified by the preceding classifier. The Random Forest model employs the technique of aggregating predictions from many decision trees generated using bootstrap sampling. The model variance was minimized by employing randomly selected subsets of features at each node. The Gradient Boosting technique was implemented by training consecutive decision trees to minimize the mistakes of preceding models [22,23,24].

### 2.7. Model Performance and Validation

To ensure a thorough assessment of model performance, the dataset was partitioned into 80% training and 20% testing subsets while maintaining the class distributions. The models were trained and evaluated using 100 different random seeds, and model stability was assessed by averaging the outcomes. The efficacy of each model was assessed and contrasted using the test set. To enhance the resilience of the prediction model and mitigate biased outcomes, the persistence technique was executed 100 times with varying random seeds, and the mean performance of these iterations was computed. Metrics, such as area under the curve (AUC), accuracy, F1 score, sensitivity, and specificity, were used to evaluate the models’ performance with a 95% confidence interval.

### 2.8. Analysis of Explainable Artificial Intelligence (XAI)

SHAP analysis, an XAI technique, was utilized to elucidate the clinical rationale behind the algorithm’s decisions and visually depict prospective lipid biomarkers’ influence. SHAP is a robust XAI technique employed to elucidate the predictions of machine learning models. This approach utilizes Shapley values from cooperative game theory to compute the contribution of each feature to model predictions in a coherent and mathematically rigorous way. SHAP analysis articulates the model output as the aggregate of each feature’s contributions, which are ascertained by the weighted average of marginal contributions computed across all potential feature combinations. In intricate machine learning models, SHAP values offer interpretability at both global (overall significance ranking of features across the entire dataset) and local (feature contributions for specific predictions) levels. This technique can uncover nonlinear correlations and intricate interactions by considering the interactions among characteristics. A key attribute of SHAP is its theoretical foundation, which ensures equitable attribution of model predictions; this characteristic provides essential aspects, such as consistency, local accuracy, and handling of missing information. SHAP values can be efficiently visualized. Numerous techniques, such as summary, dependency, and power graphs, offer a thorough comprehension of model behavior. In a clinical environment, SHAP offers valuable insights into the function of biomarkers in illness classification, assesses the significance of risk variables, and aids in treatment decision-making. This approach is a significant tool for analyzing high-dimensional omics data, facilitating the identification of critical features in complex biological systems and their association with biological processes [25,26,27,28,29].

## 3. Results

FC analysis was conducted to identify differentially expressed lipids between liver cancer and control samples. The study revealed a variety of significant alterations in lipid levels, as indicated by the FC, logarithmic FC (log_2_(FC)), adjusted *p*-values (p.adjusted), and −log_10_(p) values. Table 1 demonstrates the details of univariate statistical analysis. The differential expression (Diff. Exp.) column summarizes whether the lipid levels were upregulated or downregulated in liver cancer samples relative to the controls.

Several sphingomyelins (SMs) exhibited significant downregulation in liver cancer. SM d39:2 and SM d41:2 showed a log_2_(FC) of −0.42327 and −0.29746, respectively, with both lipids exhibiting highly significant *p*-values (p.adjusted = 0.014212), corresponding to −log_10_(p) values of 18.473. Other SMs, including SM d36:3 and SM d36:1, were also downregulated with log_2_(FC) values of −0.33284 and −0.20926, respectively.

Certain fatty acids (FAs), such as FA 14:1 (physeteric acid) and FA 22:2 (docosadienoic acid), were upregulated in liver cancer, with log_2_(FC) values of 0.3159 and 0.61134, respectively. The adjusted *p*-values for these fatty acids were 0.015341, and 0.039493, with corresponding −log_10_(p) values of 18.141 and 14.035. Furthermore, phosphatidylcholines (PCs), such as PC 34:1 and PC 32:1, were found to be upregulated, with log_2_(FC) values of 0.21334 and 0.50094, respectively. These PCs had adjusted *p*-values of 0.039493 and 0.04632, with −log_10_(p) values of 14.035 and 13.342, respectively. Of particular interest, the ceramides displayed varied expression changes. While Cer-OS d41:3 (Cer-OS d18:2/23:1) was upregulated (log_2_(FC) = 0.17604), Ceramide d41:1 was downregulated (log_2_(FC) = −0.16494), both with an adjusted *p*-value of 0.039493 and −log_10_(p) of 14.035.

**Figure 1:**
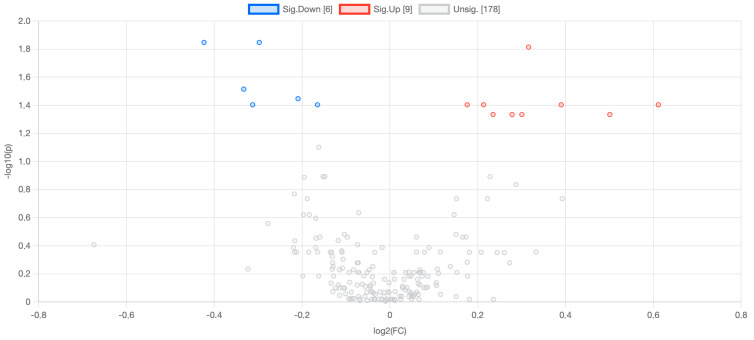
Volcano plot illustrating differentially expressed lipids. Red/blue dots denote upregulated/downregulated lipids (FDR-adjusted *p* < 0.05, |log2FC| > 0.2). Gray dots: nonsignificant. Axes labels: Log2(Fold Change) vs. −log10(*p*-value).

The volcano plot demonstrates the differential expression of lipids between liver cancer and control samples, highlighting significant findings. The *x*-axis represents the log_2_ FC, where lipids on the left are downregulated and those on the right are upregulated in liver cancer. The *y*-axis displays the −log_10_ of the adjusted *p*-value, indicating the statistical significance of these changes. Significantly downregulated lipids are marked in blue on the left, while upregulated lipids are in red on the right, with a noticeable predominance of upregulated lipids in liver cancer. Grey points near the center represent lipids with insignificant changes. The plot makes it clear that lipid metabolism is greatly changed in liver cancer, with many lipids showing significant upregulation or downregulation. This gives us important information about possible biomarkers and therapeutic targets (Figure 1).

**Figure 2:**
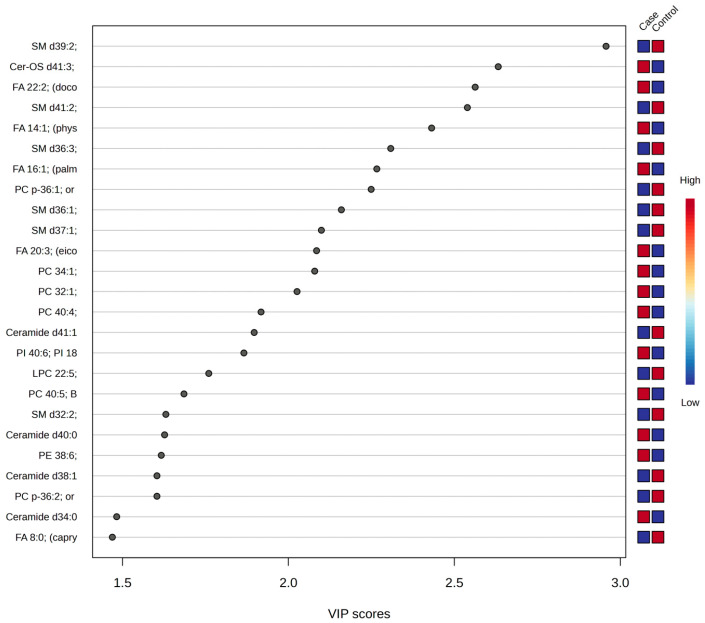
Variable importance graph for the PLS-DA model.

The PLS-DA VIP score plot highlights the most important lipids for distinguishing between liver cancer and control samples, with SM d39:2 emerging as the most influential variable, followed by Cer-OS d41:3 and FA 22:2 (docosadienoic acid). Lipids with higher VIP scores are critical in differentiating the two groups, suggesting significant molecular alterations in liver cancer (Figure 2).

Table 2 lists the candidate lipid biomarkers associated with liver cancer based on three different methods. Lipids common to the three methods were used in subsequent analyses.

Table 3 presents the performance metrics of three machine learning models—AdaBoost, RF, and Gradient Boosting—evaluated on a classification task. Among the models, AdaBoost consistently outperforms the others, achieving the highest accuracy (0.815), F1-score (0.821), sensitivity (0.852), specificity (0.778), and AUC (0.875), indicating it is the most effective model for liver cancer prediction. RF performs moderately well, with an accuracy of 0.759 and an AUC of 0.825. Gradient Boosting has the lowest performance, with an accuracy of 0.713, a specificity of 0.648, and an AUC of 0.778, suggesting it is less effective for this dataset. AdaBoost is the most robust model, showing the strongest balance between sensitivity and specificity and the highest predictive power as indicated by its AUC.

**Figure 3:**
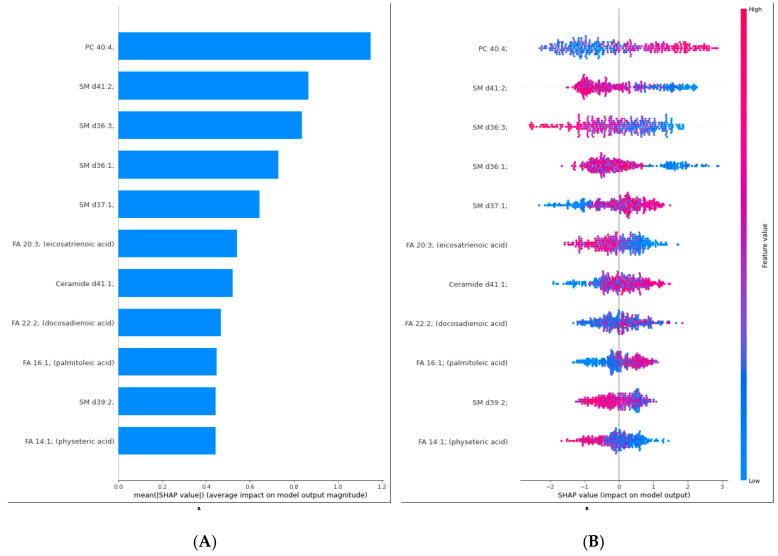
Interpretation of model prediction results with SHAP; (**A**): SHAP bar plot of Liver cancer prediction explainability; (**B**): SHAP summary plot of Liver cancer prediction explainability.

Figure 3A illustrates the mean SHAP values, quantifying each lipid feature’s average impact on the model’s output. The lipid PC 40:4 shows the highest mean SHAP value, indicating it significantly influences the model’s predictions for distinguishing between liver cancer and control samples. Other important features include SM d41:2, SM d36:3, and SM d36:1, all of which also have high mean SHAP values, highlighting their substantial contribution to the model’s decision-making process.

Figure 3B provides a detailed view of how individual SHAP values for each lipid influence the model’s output. Each point on the plot represents a single SHAP value for a particular lipid, with the color indicating the corresponding feature value (low in blue, high in red). PC 40:4 has a wide distribution of SHAP values, with high feature values generally pushing the model’s output toward a positive classification (likely indicating liver cancer). Similar patterns are observed for other lipids, such as SM d41:2 and SM d36:3. This detailed visualization confirms the significance of these lipids in the model’s predictions and provides insights into how variations in lipid levels impact the classification outcomes.

## 4. Discussion

The findings of this study indicate significant alterations in lipid profiles among liver cancer patients when compared with control groups. These alterations suggest that lipid profiles could serve as potential diagnostic and therapeutic tools, though further validation is required. However, the big picture is also well aligned with the main goal of identifying the molecular markers that are important for further progress in understanding liver cancer and developing related therapies. The present work will also help to improve the understanding of simple biochemical processes involved in cancer and reveal novel biomarkers and targets for molecular diagnosis, and the therapy of malignant diseases. The differential expression of several lipid species provides a clue to their potential as biomarkers and opens up prospects for analyzing the reprogramming of energy metabolism in cancer cells. Additionally, the machine learning and SHAP approaches used in this study add credibility to such conclusions and demonstrate the significance of analyzing the present research issue from a data perspective [30,31]. This research paper investigates the biological relevance of these findings, their correlation with previous research, and the applications of these findings to the development of subsequent investigations and interventions for liver cancer. Additionally, tree-based models were selected over deep learning due to their interpretability and robustness in high-dimensional and small-sample lipidomic data.

Lipidomic data are used for the discovery of biomarkers and the development of predictive models in a few studies. It was determined from a medical study that there were 85 phospholipids that are different in HCC from normal tissues, and LPG was associated with an increased risk of mortality in HCC patients [32]. It was found that diacylglycerol (DAG) and ceramide play a crucial part in Wnt/β-catenin signaling and tumor growth and proliferation in studies in HCC cell lines [33].

A clinical use of the SALDI MS lipidomic profiling method based on 98.3% diagnostic accuracy for liver cancer was shown in a clinical study [34]. In another study, the Fast Evaporative Ionization Mass Spectrometry method was used to compare lipid profiles in HCC tissues, which showed 100% sensitivity for real-time diagnosis [34]. Liu et. al. [35] reported that HCC treatment can be through the inhibition of specific lipid metabolic pathways like those of phosphatidylcholine and ceramide. Nevertheless, they have undertaken such studies on the lipid biomarkers using classical statistical or machine learning methods. However, to the best of our knowledge, the current study is the first paper to develop an interpretable approach based on XAI using lipidomic panel data.

The downregulation of sphingomyelins (SMs), such as SM d39:2 and SM d41:2, has been seen in liver cancer. This underscores the critical role of lipid metabolism dysregulation in cancer progression. The sphingomyelins are an important part of the membranes of cells, so they help send signals and control apoptosis [7]. Our study consistently downregulated them, as evidenced by statistically significant fold changes and corrected *p*-values. This could be due to slower biosynthesis rates and faster breakdown patterns, which higher sphingomyelinase levels could cause. This situation might change the metabolism of cancer cells, which would help them stay alive longer by changing the membrane lipid profile to stop apoptosis. These results are similar to what the studies [36,37] have already reported about cancers. This supports the idea that sphingolipid dysregulation is one of the three main signs of liver cancer. Most importantly, it is noteworthy whether these particular SMs may serve as a new therapeutic target, since their alterations could indeed reverse the cancer process. Therefore, future studies should employ a broad approach to examine the validity of these mechanisms in tumorigenesis and as biomarkers.

The rise in fatty acids like FA 14:1 (physeteric acid) and FA 22:2 (docosadienoic acid) is very important because it shows how important they are for making energy. The fluidity of the membrane and the signaling pathways are crucial for the survival and growth of cancer cells. Higher amounts of these unsaturated fatty acids in liver cancer samples may mean that more de novo lipogenesis is happening. This is a key sign of cancer because it means that the body’s metabolism is changing in ways that help the tumor grow quickly [38]. This discovery supports the idea that cancer cells depend on lipid biosynthesis to produce structural elements and energy reserves. This is especially true when the availability of nutrients is limited. Along with fatty acids, the big rise in phosphatidylcholines (PCs), such as PC 34:1 and PC 32:1, shows that phospholipid metabolism has changed in a big way. Phosphatidylcholines play a vital role in the formation of membranes and cell signaling. Their increased levels may result from the greater need for membrane production in cancer cells actively dividing. This observation supports earlier research [39,40] emphasizing that abnormal phospholipid metabolism is crucial in cancer development. Additionally, the dual functions of fatty acids and PCs in the progression of liver cancer indicate that focusing on these metabolic pathways may offer therapeutic advantages. Future research should explore the potential of modulating key enzymes involved in fatty acid synthesis and PC production. Key enzymes like choline kinase and fatty acid synthase (FASN) might be able to stop tumors from growing or make current treatments work better. These interventions might also shed light on whether these metabolic changes are simply a consequence of tumorigenesis or if they play a crucial role in driving it forward. The results add to the growing body of evidence that changes in lipid profiles are important for cancer metabolism and point out a possible way to find biomarkers and make drugs [41].

The different levels of ceramides with Cer-OS d41:3 upregulated and ceramide d41:1 downregulated show how complicated ceramide metabolism is in liver cancer. Ceramides functioning as bioactive lipids are crucial in regulating cellular stress responses, apoptosis, and autophagy [5]. The increase in Cer-OS d41:3 might act as a compensatory response to inhibit tumor growth by promoting apoptosis and controlling excessive proliferation. Conversely, the downregulation of ceramide d41:1 may indicate its preferred role in metabolic pathways promoting tumor progression, such as glycosphingolipid synthesis, associated with enhanced survival and invasiveness in cancer cells. Because ceramides have two functions, it shows how complex their role is in tumor biology. Depending on the cell context and the specific ceramide species present. They can act as both suppressors and facilitators. This observation is consistent with the study’s findings [42], highlighting the context-dependent nature of ceramides in cancer. The insights presented here strongly justify additional research into ceramide metabolism as a potential therapeutic target. This is especially important for finding ways to change pro-apoptotic ceramides to stop tumor growth while also stopping mechanisms that might help the tumor stay alive [5,43]. AdaBoost, among all the models trained, encompasses the utmost commendable performance in accuracy, sensitivity, and AUC. This additionally endorses the significance of new machine learning methods in improving diagnosis. The in-depth SHAP analyses explain that PC 40:4 plays an indispensable role in distinguishing liver cancer from controls. A determined high influence of PC 40:4 on model predictions suggests its applicability as a potential biomarker in diagnosing liver cancer, which is as intended by this study that aims at using lipidomic data for clinical use. PC 40:4 has been previously implicated in phospholipid remodeling pathways associated with cancer progression. Its role in liver cancer remains underexplored, but its elevated levels in our study may suggest increased membrane biosynthesis and metabolic adaptation in malignant cells [44]. Moreover, the broader SHAP value distributions demonstrate how the individual model making decisions is affected by the lipid features, which are reaffirmed alongside the aim of such decisions—promoting the idea of precision medicine. This observation, alongside the findings, was in line with and further endorsed the study’s findings by highlighting PC species as the most vital constituents in cancer diagnostics. The combination of explainable AI methods not only achieves the objective of efficiently selecting important lipids but also provides model interpretability, which increases the clinical acceptance of the approach [45,46].

The significant alterations in lipid metabolism highlighted in this research provide insights into the functioning of liver cancer and aid in achieving the research objectives concerning enhancing the diagnosis and treatment procedures. Changing sphingomyelins, fatty acids, and phosphatidylcholines shows a deep reprogramming of metabolism that is needed for long-term survival and shows how these lipid changes may be used as clinical biomarkers. By interpreting such metabolic alterations, this research integrates basic lipidomic research with its applications. In this manner, we deepen our understanding of the biology of liver cancer. These findings also facilitate the design of innovative lipid-based diagnostic and therapeutic agents that may affect altered metabolic pathways in liver cancer. This is consistent with the overarching aim of enhancing the quality of care for cancer patients through precision medicine [44,47].

In addition to these outcomes, the diagnosis, treatment, and prognosis of liver cancer are greatly improved by AI technologies and image processing. The radiomics analyses supported by the AI are very accurate, at least with respect to early diagnosis and tumor classification from the data derived from the CT and MRI images, to help in personalized treatment planning. Traditional surgical systems employ augmented reality and robotic surgery systems to increase treatment success, but deep learning models help predict the prognosis by estimating the treatment response and recurrence risk. Nevertheless, more clinical validation is required due to the standardization of the data and algorithm generalizability limits [48]. Butyrylcholinesterase (BChE) is an enzyme synthesized in the liver and plays a role in the hydrolysis of various ester compounds. In the literature, BChE has been used as a marker of the risk of developing surgical site infections (SSIs) and septic complications in patients undergoing colorectal surgery [49,50].This finding points to the potential role of BChE in immune response and inflammation processes. In liver cancer, it is thought that BChE levels may be a prognostic indicator. In particular, low BChE levels may indicate impaired liver function and advanced stages of the disease. However, more comprehensive and up-to-date studies are needed to determine the exact role of BChE in liver cancer and its place in clinical practice.

In future research, it will be necessary to test these findings on large populations and to comprehend the pathophysiological mechanisms of lipid alterations in the context of liver cancer. Lipidomic alterations in liver cancer should also be studied in combination with proteomic and genomic data so that a clearer picture of the altered metabolism of the liver in this disease can be obtained. Longitudinal studies of lipid changes during disease and treatment will be useful to determine their prognostic value. However, this study has several limitations, even if it brings new knowledge concerning lipid metabolism disorders in liver cancer. First, the generalizability of using a relatively small sample size may be questioned upon testing this study in larger heterogeneous populations. Furthermore, the cross-sectional nature of the study design limits the ability to infer the causality of the observed lipid metabolism changes and the advance of liver cancer. In this case, applying lipidomic profiling alone without the transcriptomic or proteomic data does not allow one to fully explore the interplay of metabolic pathways. Furthermore, although the development of machine learning models has improved the diagnostic accuracy of lipid biomarkers. We cannot exclude the possibility of overfitting due to the limited data set size, and there are ongoing studies that aim to overcome these limitations, particularly using longitudinal designs.

## 5. Conclusions

This study demonstrates that liver cancer induces significant alterations in lipid metabolism, suggesting that specific lipids may serve as valuable biomarkers for both diagnosis and treatment. This study demonstrates the diagnostic accuracy of lipidomics when combined with advanced statistical analyses and machine learning models. This finding aligns with the broader goal of advancing precision medicine. These findings lay a robust groundwork for subsequent investigations into the intricate metabolic pathways implicated in liver cancer. Such efforts could catalyze the development of innovative diagnostic tools and targeted therapeutic strategies. This could ultimately lead to an advancement in patient care and treatment efficacy.

## Figures and Tables

**Table 1:** Results of univariate statistical analysis.

Name	FC	log_2_(FC)	p.adjusted	−log_10_(p)	Diff. Exp.
SM d39:2;	0.74573	−0.42327	0.014212	18.473	Down
SM d41:2;	0.81368	−0.29746	0.014212	18.473	Down
FA 14:1; (physeteric acid)	12.448	0.3159	0.015341	18.141	Up
SM d36:3;	0.79397	−0.33284	0.030552	1.515	Down
SM d36:1;	0.86498	−0.20926	0.035719	14.471	Down
FA 22:2; (docosadienoic acid)	15.277	0.61134	0.039493	14.035	Up
FA 16:1; (palmitoleic acid)	13.105	0.39014	0.039493	14.035	Up
SM d37:1;	0.80521	−0.31256	0.039493	14.035	Down
PC 34:1;	11.594	0.21334	0.039493	14.035	Up
Cer-OS d41:3; Cer-OS d18:2/23:1;	11.298	0.17604	0.039493	14.035	Up
Ceramide d41:1;	0.89196	−0.16494	0.039493	14.035	Down
PC 32:1;	14.151	0.50094	0.04632	13.342	Up
PC 40:4;	12.316	0.30051	0.04632	13.342	Up
FA 20:3; (eicosatrienoic acid)	12.128	0.2783	0.04632	13.342	Up
PI 40:6; PI 18:0-22:6;	11.769	0.23502	0.04632	13.342	Up

**Table 2:** Important biomarker candidate lipids in liver cancer according to different approaches.

Elastic Net Feature Importance	PLS-DA VIP Score	Fold Change
1_PG 17:0/17:0; iSTD	SM d39:2;	SM d39:2;
Ceramide d41:1;	Cer-OS d41:3; Cer-OS d18:2/23:1;	SM d41:2;
Ceramide d42:2; B	FA 22:2; (docosadienoic acid)	FA 14:1; (physeteric acid)
Ceramide d43:1;	SM d41:2;	SM d36:3;
FA 14:0; (myristic acid)	FA 14:1; (physeteric acid)	SM d36:1;
FA 16:0; (palmitic acid)	SM d36:3;	FA 22:2; (docosadienoic acid)
FA 18:1; (oleic acid)	FA 16:1; (palmitoleic acid)	FA 16:1; (palmitoleic acid)
FA 18:2; (linoleic acid)	PC p-36:1; or PC o-36:2;	SM d37:1;
LPC 18:2;	SM d36:1;	PC 34:1;
PC 34:3;	SM d37:1;	Cer-OS d41:3; Cer-OS d18:2/23:1;
PC 36:1;	FA 20:3; (eicosatrienoic acid)	Ceramide d41:1;
PC 38:2;	PC 34:1;	PC 32:1;
PC 40:4;	PC 32:1;	PC 40:4;
PC 36:2;	PC 40:4;	FA 20:3; (eicosatrienoic acid)
SM d36:1;	Ceramide d41:1;	PI 40:6; PI 18:0-22:6;
SM d37:1;	PI 40:6; PI 18:0-22:6;	
SM d39:2;	LPC 22:5;	
SM d40:2; B	PC 40:5; B	
SM d40:1;	SM d32:2;	
SM d41:2;	Ceramide d40:0;	
FA 20:3; (eicosatrienoic acid)	PE 38:6;	
FA 22:2; (docosadienoic acid)	Ceramide d38:1;	
FA 16:1; (palmitoleic acid)	PC p-36:2; or PC o-36:3;	
FA 14:1; (physeteric acid)	Ceramide d34:0;	
SM d36:3;	FA 8:0; (caprylic acid)	

**Table 3:** Performance metrics results of tree-based machine learning models for liver cancer detection.

Metric	AdaBoost	Random Forest	Gradiant Boosting
Accuracy	0.815 (0.742–0.888)	0.759 (0.679–0.84)	0.713 (0.628–0.798)
F1-Score	0.821 (0.749–0.894)	0.772 (0.693–0.851)	0.73 (0.647–0.814)
Sensitivity	0.852 (0.729–0.934)	0.815 (0.686–0.907)	0.778 (0.644–0.88)
Specificity	0.778 (0.644–0.88)	0.704 (0.564–0.82)	0.648 (0.506–0.773)
AUC	0.875 (0.815–0.934)	0.825 (0.760–0.890)	0.778 (0.713–0.844)

## Data Availability

The raw data supporting the conclusions of this article will be made available by the authors on request.
